# Driving Style Recognition Based on Electroencephalography Data From a Simulated Driving Experiment

**DOI:** 10.3389/fpsyg.2019.01254

**Published:** 2019-05-29

**Authors:** Fuwu Yan, Mutian Liu, Changhao Ding, Yi Wang, Lirong Yan

**Affiliations:** ^1^Hubei Key Laboratory of Advanced Technology for Automotive Components, School of Automotive Engineering, Wuhan University of Technology, Wuhan, China; ^2^Hubei Collaborative Innovation Center for Automotive Components Technology, School of Automotive Engineering, Wuhan University of Technology, Wuhan, China

**Keywords:** driving style, EEG, driving behavior, driving data, K-means, support vector machine

## Abstract

Driving style is a very important indicator and a crucial measurement of a driver's performance and ability to drive in a safe and protective manner. A dangerous driving style would possibly result in dangerous behaviors. If the driving styles can be recognized by some appropriate classification methods, much attention could be paid to the drivers with dangerous driving styles. The driving style recognition module can be integrated into the advanced driving assistance system (ADAS), which integrates different modules to improve driving automation, safety and comfort, and then the driving safety could be enhanced by pre-warning the drivers or adjusting the vehicle's controlling parameters when the dangerous driving style is detected. In most previous studies, driver's questionnaire data and vehicle's objective driving data were utilized to recognize driving styles. And promising results were obtained. However, these methods were indirect or subjective in driving style evaluation. In this paper a method based on objective driving data and electroencephalography (EEG) data was presented to classify driving styles. A simulated driving system was constructed and the EEG data and the objective driving data were collected synchronously during the simulated driving. The driving style of each participant was classified by clustering the driving data via K-means. Then the EEG data was denoised and the amplitude and the Power Spectral Density (PSD) of four frequency bands were extracted as the EEG features by Fast Fourier transform and Welch. Finally, the EEG features, combined with the classification results of the driving data were used to train a Support Vector Machine (SVM) model and a leave-one-subject-out cross validation was utilized to evaluate the performance. The SVM classification accuracy was about 80.0%. Conservative drivers showed higher PSDs in the parietal and occipital areas in the alpha and beta bands, aggressive drivers showed higher PSD in the temporal area in the delta and theta bands. These results imply that different driving styles were related with different driving strategies and mental states and suggest the feasibility of driving style recognition from EEG patterns.

## Introduction

Driving style generally refers to the way a driver prefers to or habitually drives the car (Motonori et al., 2007; Martinussen et al., 2014). It is based on a compilation of cognitive, emotional, sensory and motor factors occurring over space and time (Lin et al., 2006a; Yang et al., 2018). Rather than events that happen at any given moment, the driving style, as describe by internal states of the human, seems to be less informative than the measurable driving behaviors. However, the driving style does have some relationship with the driving behaviors. Previous studies have suggested that the driving style can be classified into three types: Aggressive type, Moderate type, and Conservative type (Chu et al., 2017; Deng et al., 2017; Li et al., 2017; Palat et al., 2019). Different driving styles can result in different kinds of behaviors and actions of the drivers and vehicles. The Aggressive driving style is usually associated with faster speed, acceleration, and larger steering wheel rotation angle and angular velocity, whereas a Conservative driving style is usually associated with longer space headway, larger angle of the brake pedal, and longer deceleration. The moderate driver drives with relative steady motions that are neither too conservative nor too aggressive (Lu, 2011; Hooft van Huysduynen et al., 2018; Yang et al., 2018). In general, driving style is affected by personality, and the physical and mental state of the driver, and externally manifested as driving behaviors. It is noted that the driver with dangerous driving styles would not necessarily, but quite possibly, conduct the dangerous driving behaviors, hence the driving style would be a very important indicator and a crucial measurement of a driver's performance and ability to drive in a safe and protective manner. If the driving styles can be recognized by some appropriate classification methods, much attention could be paid to the drivers with dangerous driving styles. The driving style recognition module can be integrated into the advanced driving assistance system (ADAS), which integrates different modules to improve driving automation, safety and comfort, and then the driving safety could be enhanced by pre-warning the drivers or adjusting the vehicle's controlling parameters when the dangerous driving style is detected. Therefore, driving style recognition has been intensively investigated in the field of transportation and automobile safety.

Over the years, researchers have developed a number of driving style recognition methods based on questionnaire data. For example, a quantitative method based on the Driving Behavior Questionnaire (DBQ) was proposed to classify driving styles and investigate the distinction among three aberrational driving behaviors, i.e., violations, errors and lapses. Violations are the intended acts that a person is most likely aware of, such as speeding or running a red light. People know clearly the consequences but still conduct the violations intentionally. Errors are acts that fail at the planned and intended outcome due to misjudgments, such as abrupt braking. Lapses are unintentional behaviors performed because of poor attention or memory deficits, such as missing the motorway exit (Reason et al., 1990). Lajunen and Summala (1995) constructed the Driving Skill Inventory(DSI) to measure the skill and safety-motive dimensions (transient motivational, personality and attitudes toward safety and traffic) in drivers' self-assessments of their driving styles and abilities. Furthermore, Motonori et al. (2007) developed the Driving Style Questionnaire(DSQ) to specifically classify driving styles and demonstrated validity using a car-following experiment. A hybrid model based on DBQ and DSI was proposed to classify drivers into sub-groups based on their driving styles and driving skills (Martinussen et al., 2014). Deng et al. (2018) applied DBQ-based driving styles to curve safety speed model to determine the theoretical curve safe speed, and the results indicated the new model could not only prevent the risks of rollover and sideslip during turning, but also could adapt to the driver's driving style. Although promising results were obtained, the questionnaire investigation was prone to the subjective factors of the researchers and the participants. In addition, this approach could not provide dynamic real-time identification and prevention of dangerous behaviors and hence, is not useful during actual driving.

Objective driving data such as vehicle speed and acceleration were also utilized as the data sources for driving style recognition. In actual driving experiments, these driving data were collected by in-vehicle sensors, transported by the vehicle's Controller Area Network (CAN)-Bus, and then analyzed to identify driving style by using the pattern recognition method (Choi et al., 2007; Ly et al., 2013). Due to the complexity and low repeatability of the actual driving experiment, a number of researchers chose to conduct experiments on simulated driving platforms (Hooft van Huysduynen et al., 2018; Yang et al., 2018). In contrast to the questionnaire studies, driving style recognition based on objective data is not prone to subjectivity, and the online real-time analysis can be achieved. But these objective driving data mostly reflect the behaviors of the vehicle, which are the external or resultant outcome of the driver's driving style. As noted above, dangerous driving styles are more likely to trigger dangerous behaviors, but not necessarily. The purpose of driving style recognition is to evaluate the possibility of the occurrence of dangerous driving behaviors and then introduce prevention measures. It may be insufficient to build up the temporal and causal relationship between driving behavior and driving style only by using objective driving data. More direct and precise evaluation of the driver's state might be helpful.

A number of studies have utilized electroencephalography (EEG) to identify dangerous driving states, such as fatigue and distraction (Chuang et al., 2015; Hajinoroozi et al., 2016; Belakhdar et al., 2018; Guo et al., 2018; Ma et al., 2018), driving behaviors, such as emergency braking (Haufe et al., 2011), speeding (Lutz et al., 2008) and turning (Taghizadeh-Sarabi et al., 2013), and driving styles, such as car-following and obstacle-dodging (Lin et al., 2006b; Yang et al., 2018). Specifically, some researchers classify and assess the driver's behavior and style based on the amplitude and power spectral density information of α, β, δ, and θ bands of EEG signals. For example, Lin et al. (2006b) used the power spectrum analysis to investigate the correlation between driving style and brain activities revealed by EEG, and found power difference at 10 Hz and 20 Hz between aggressive and conservative drivers. Taghizadeh-Sarabi et al. (2013) extracted the absolute power of these four bands by Fast Fourier Transforms (FFT) to assess the driver's cognitive responses when turning left and right. Yang et al. (2018) combined the amplitude and the power spectral density to classify the driver's driving skill and driving style. As mentioned above, driving style is related with cognitive, emotional, sensory and motor factors, and EEG patterns across different brain areas can effectively reflect these factors. Compared with the moderate and conservative drivers, the drivers with the aggressive driving styles had more intensive emotion fluctuations and difficulties in emotion regulation (Trógolo et al., 2014; Zhang et al., 2016), which was associated with the delta and theta power in the temporal area (Knyazev et al., 2008, 2009). The aggressive drivers were more likely to engage in aberrational driving behaviors (Reason et al., 1990; Martinussen et al., 2014; Lee and Jang, 2017), which was resultant from the poor cognitive states and cognitive failures (Wickens et al., 2008) and related with the theta/beta ratio of the EEG signal in the frontal area (Angelidis et al., 2018; Puma et al., 2018). Besides, some studies suggested that high beta power in the parietal area was associated with the pro-active driving state, which was related with a better anticipation and active use of ongoing information, and a more proactive planning of future responses (Tao et al., 2010; Garcia et al., 2017; Getzmann et al., 2018). Compared with the traditional driving data and the questionnaire data, EEG shows several advantages in driving style recognition. Specifically, EEG data has a time resolution of milliseconds, allowing for more accurate real-time classification; EEG can provide physiological data and emotional data, without disturbing driving behaviors (Yang et al., 2018). More importantly, EEG data is not only an objective, but also a direct reflection of the driver's cognitive status, which can be predictive of future unsafe driving behaviors. Therefore, EEG has great potential in driving style recognition.

The aim of this study was to develop a driving style recognition method based on EEG data. A simulated driving system was constructed, the driving data and the EEG data were collected synchronously and then analyzed by machine learning algorithms. Our results demonstrated the strong correlations between driving style as measured by driving data and EEG patterns.

## Materials and Methods

### Experiment and Participants

#### Participants

Twenty-three healthy participants with a driver's license, 21 males and 2 females, with a mean age of 23.6 ± 1.6 years and average driving experience of 2.9 ± 1.7 years, were recruited and participated in the simulated driving experiment. This study was carried out in accordance with the recommendations of the ethical review committee of Wuhan University of Technology with written informed consent in accordance with the Declaration of Helsinki from all participants. The protocol was approved by the ethical review committee of Wuhan University of Technology.

#### Driving Scenario and Task

**Figure 1:**
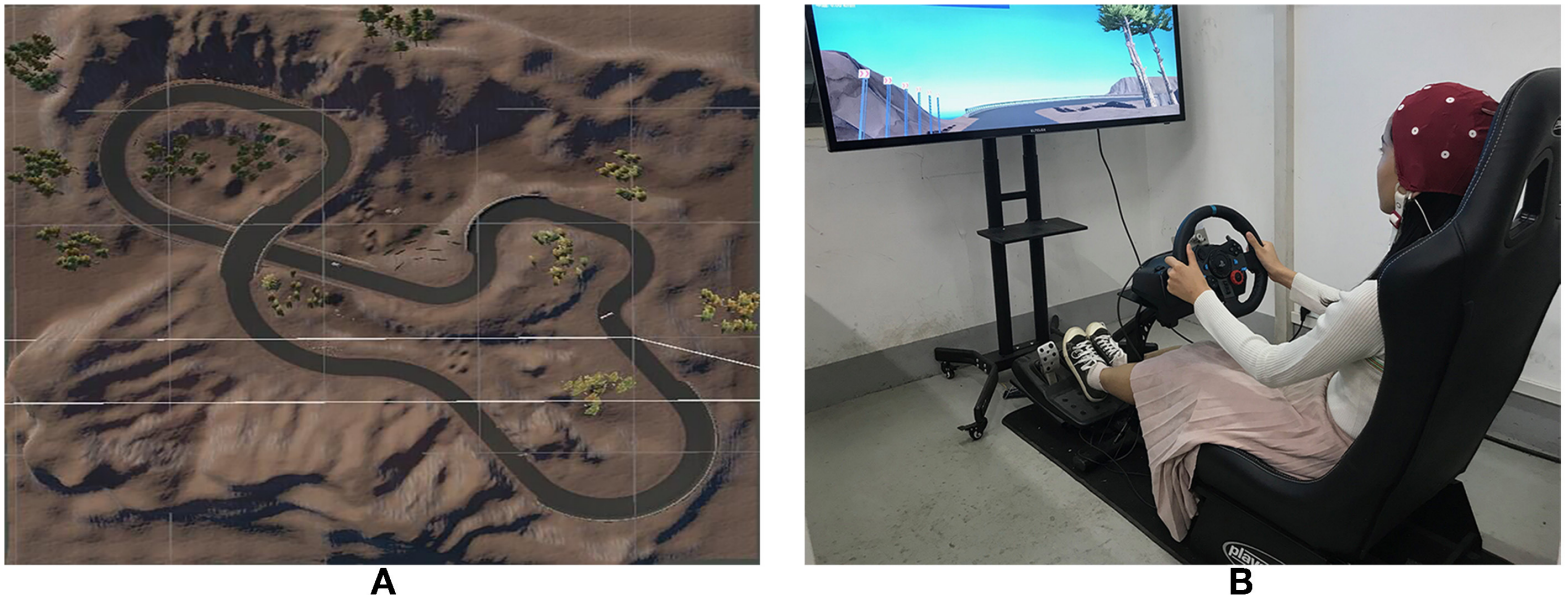
Simulated driving system. **(A)** driving track, **(B)** simulated driving platform. The participant has provided written consent for the publication of this image.

The driving scenario was designed based on Unity 3D (Unity Technologies, USA). Previous studies demonstrated the coupling between turning and driving styles (Ly et al., 2013; Choi et al., 2017; Deng et al., 2018), brain dynamics (Garcia et al., 2017). Hence a seven-kilometer circular road containing two consecutive S-shaped curves, two curved roads with a radius of 20 m and seven other curves in a montanic scenery was applied (Figure 1A). There was a left or right turn sign before each curve and some simulated vehicles were placed on the road. Each participant was asked to start a simulated compact car at the starting line and drive along the circular road. Four laps of driving was taken as a driving task and each participant completed two to four tasks. After each task, they took a break for a few minutes to avoid driving fatigue. The participants were asked to pay attention to the traffic signs and the real-time speed of the vehicle, and drive according to their driving habits and styles in daily life. The speed limit was 60 km/h. The driving task was performed using a simulated driving system including a driving simulator (G29, Logitech Inc., Fremont, CA) consisting of a steering wheel, a full-size driving seat, a stick shift and three pedals, and a 50-inch screen (Figure 1B). All participants were given at least half an hour to adapt to the simulator and the driving task to ensure they were all proficient in driving in the simulator.

#### Data Acquisition

When the participants were performing the driving task, their EEG signals as well as the state of the steering wheel were collected. EEG signals were recorded continuously using a 16-channel (Fz, Cz, Pz, T6, T5, C4, C3, F8, T4, T3, O2, O1, P4, P3, Fp1, Fp2) Biopac MP150 system (Biopac, Goleta, USA) with a 10–20 system layout at a sampling rate of 1000 Hz. The left earlobe was used as the reference. A photoelectric encoder was tightly coupled with the steering wheel by a synchronous belt so that the rotation of the steering wheel drove the axle of the photoelectric encoder to rotate synchronously. A circuit based on the photoelectric encoder was developed using Arduino microcontrollers to acquire the steering wheel's angular velocity, rotation angle and angular acceleration at a transmission rate of 128000Bd. During driving, the number of collisions and the number of lane excursions were recorded.

### Data Analysis

**Figure 2:**
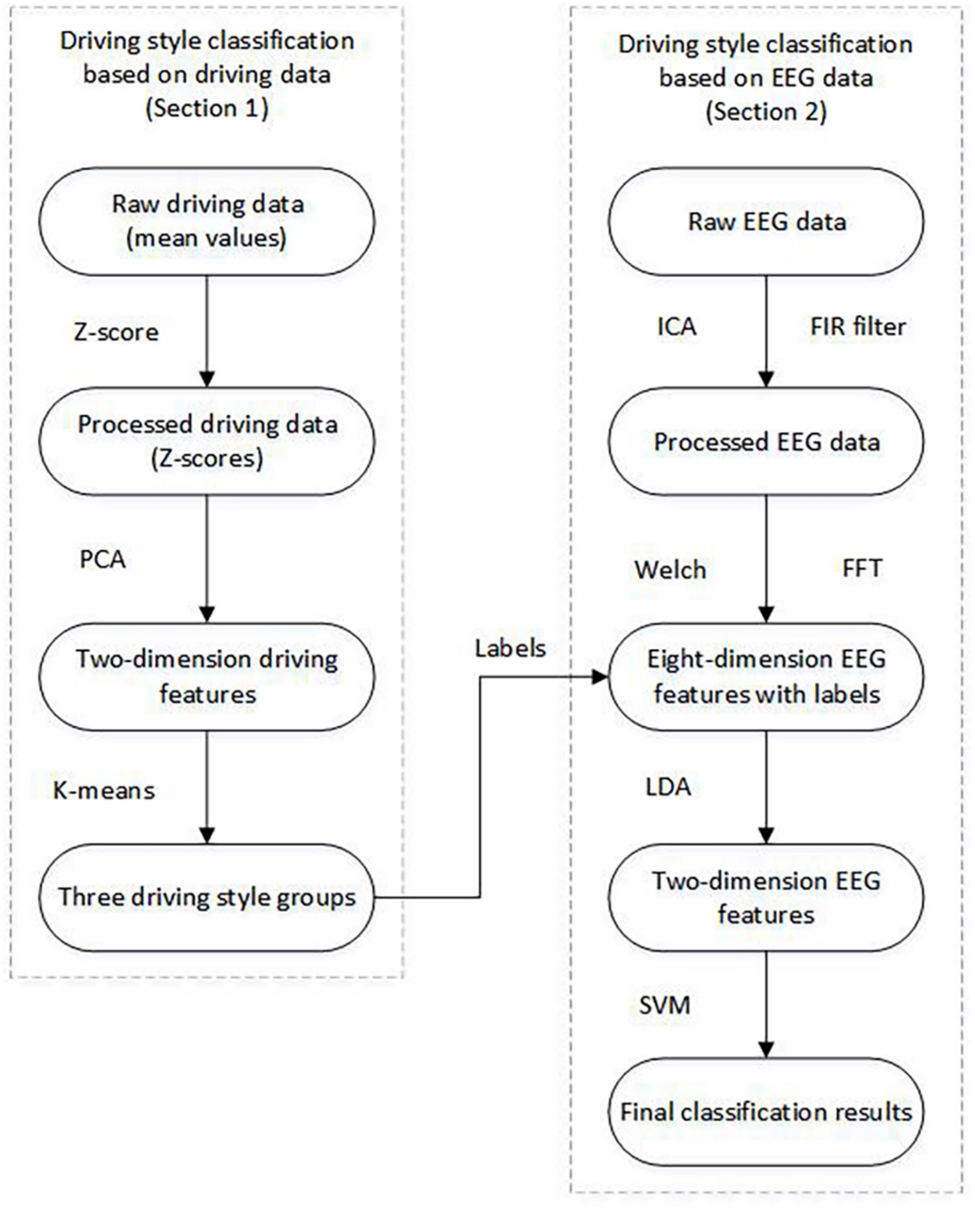
Flow chart of the EEG-based driving style recognition.

A driving style recognition schema was proposed (Figure 2). The schema contained two sections: driving performance data-based recognition and EEG-based recognition. In section Introduction, the driving data was considered as the measure for classifying the driving style and the participants were divided into different groups. In section Materials And Methods, the EEG data, combined with the classification results of section Introduction as labels, were utilized to establish the Support Vector Machine (SVM) model to recognize the driving styles.

#### Driving Data Analysis

Seven variables including the steering wheel's rotation angle, angular velocity, angular acceleration, total driving time, vehicle velocity and the number of accidents (collision) and aberrations (lane excursion) were selected as the driving data for further analyses. All participants completed the violation-item and error-item of the DBQ, and were divided them into three driving style groups according to their scores. Firstly, the seven key variables of the 75 tasks were averaged and standardized using the *Z*-score method. These 7-dimension *Z*-scores were reduced to 2-dimensions using Principal Component Analysis (PCA) (Jolliffe and Cadima, 2016). A low-dimensional matrix **M**_**L**_ (${\text{M}}_{\text{L}}\in {\text{R}}^{75\times 2}$) was obtained and then clustered by the *K*-means clustering method. The K-means algorithm is an unsupervised learning method aiming to classify **n** samples into **K** clusters by minimizing the squared error over all **K** clusters (Bolin et al., 2014; Yang et al., 2018). The K-means algorithm can be formulated as follows.

(1) Initialization. Specify the number of clusters ***K***, form the initial cluster centroids (**μ**_**k**_ as the centroid for cluster **C**_**k**_) either by using random selection or through pre-specification of cluster centroids by the researcher, and assign each observation to the nearest cluster.
(2) Calculate the squared Euclidean distance (ESS) (Equation 1) based on the current cluster.

(1) $$ESS=\sum_{k=1}^{K}\sum_{X_{i}\in C_{k}}{\left\|X_{i}-\mu_{k}\right\|}^{2}$$

where **X**_**i**_ is a observation of cluster **C**_**k**_.

(3) Reassign each observation to the cluster whose centroid is the nearest.
(4) Update the cluster centroids based on the new observation clusters.
(5) Repeat steps 2–4 until there is no further reassignment of the observations (i.e., each observation is in the cluster with the nearest centroid and ESS is minimized).

The number of clusters ***K*** can either be specified according to the experience of the researcher, the priori knowledge of the data, or the clustering quality assessment indicators such as Calinski-Harabasz score (Łukasik et al., 2016), Silhouette Coefficient (Luan et al., 2012), etc. We utilized Calinski-Harabasz score and computed it as follows:

$$s\left(k\right)=\;\frac{tr(B_{k})}{tr(W_{k})}\frac{m-k}{k-1}$$

where **m** is the number of training samples, **k** is the number of clusters, **B**_**k**_ is the covariance matrix between clusters, **W**_**k**_ is the covariance matrix within a cluster, **tr** is the trace of matrix.

***K*** that maximizes the criterion is chosen.

#### EEG Data Analysis

Firstly, all EEG data was denoised and preprocessed. Secondly, Fast Fourier transformation (FFT) and the Welch method were utilized to extract the EEG features, and then Linear Discriminant Analysis (LDA) was utilized to generate the core EEG features. These core EEG features were utilized to train the SVM model. Finally, the classification performance was evaluated using a leave-one-subject-out validation method. The detailed procedures are as follows.

(1) EEG data preprocessing

Because EEG signals are weak and easily contaminated by eye movements and muscular tension, it is necessary to remove the noise from the original signals. EEGLAB (Delorme and Makeig, 2004) and MATLAB (v.2016a; MathWorks, USA) were utilized for preprocessing.

The whole EEG data acquired in each task was down-sampled to 512Hz. Because the driver's driving state is closely related to the four EEG frequency bands: delta (δ: 0.5–4 Hz), theta (θ: 4–8 Hz), alpha (α: 8–13 Hz), and beta (β:13–30 Hz) (Khushaba et al., 2011; Li et al., 2012; Lin et al., 2014; Ma et al., 2018), a bandpass Finite Impulse Response (FIR) filter (0.5–30 Hz) was applied to the EEG data and the information in these four frequency bands was retained. Independent Component Analysis (ICA) was utilized to decompose the filtered EEG data into several components and the components caused by artifacts such as eye movements, blinking and muscular tension were identified based on ADJUST (Mognon et al., 2011), an EEGLAB plugin, and then removed (Akhtar et al., 2012; Ma et al., 2018). The bad channels were detected and replaced by the average of the two neighboring channels. Finally, the EEG data was re-referenced to the average reference to reduce the forward model error of each channel and baseline corrections were performed to eliminate the noise caused by spontaneous brain activity.

(2) EEG features extraction

The features of the EEG data in the frequency domain were extracted. The amplitude of the EEG signal in δ, θ, α, and β bands were obtained by using FFT, and the power spectral densities (PSDs) in these four bands were estimated using the Welch's method (Upadhyay et al., 2012).

Each participant's FFT and PSD features were integrated to generate an 8-dimensions feature vector, along with the driving style label. Then, the feature vectors were reduced to 2-dimensions using LDA for simplifying calculations in the next SVM training process and improving the final classification accuracy. Unlike PCA, LDA is a supervised dimension reduction method which needs the labeled information. It projects the original data into a low-dimensional space by maximizing the between-class distance and minimizing the within-class distance (Martinez and Kak, 2001; Yuan and Tao, 2015).

(3) EEG data classification via SVM

SVM is a supervised learning model that is commonly used for pattern recognition, classification, and regression analysis. The core content of SVM is creating hyperplanes that separate the data points of a binary classification problem. Assuming train data in the form of {(**S**_**1**_**,** **y**_**1**_)**,** (**S**_**2**_**,** **y**_**2**_)**,** **…,** (**S**_**n**_**,** **y**_**n**_)}, where **S**_**i**_ is a train sample and **y**_**i**_ is the label of **S**_**i**_**,** **y**_**i**_**∈**{**−1,** **1**}. In this SVM model, all labels were acquired based on the driving data clustering (section Driving Data Analysis), where “**−1**” represented “the Conservative driving style” and “**1**” represented “the Aggressive driving style”. The separating hyperplane can be formulized as:

(2) W·S+b=0

where **W** is the vector of the separating hyperplane, and $\frac{\text{b}}{\left||\text{W}|\right|}\;$is offset of the separating hyperplane from the origin along vector **W**. The linear SVM utilizes two parallel hyperplanes (**W · S+b= ± 1**) to divide the train data points into two groups. The train data points in the two parallel hyperplanes are called “support vector.” The distance between the two parallel hyperplanes is $\frac{2}{\left||\text{W}|\right|}$, which is called “margin.” To search for a best separating hyperplane, the “margin” needs to be maximized, or ||**W**||^**2**^ needs to be minimized as follows:

(3) $$\min\;\frac{1}{2}{\left\|W\right\|}^{2}$$

(4) $$subject\;to\;y_{i}\left(W\cdot S_{i}+b\right)\geq 1\;i=1,2,\ldots ,n$$

The Lagrange method is utilized to obtain **W** and **b** as the key parameters of the optimal hyperplane. For multi-classification problems, the core idea is to transform a single multiple classification problem into multiple binary classification problems (Duan and Keerthi, 2005), there are two methods: (1) One-Versus-Rest (OVR), Building binary classifiers that distinguish between one of the labels and the rest; (2) One-Versus-One (OVO), Building binary classifiers that distinguish between every pair of classes. In this paper, we used OVO to perform the classification. A leave-one-subject-out cross validation and the F-measure were utilized to evaluate the performance of the classification.

## Results

### Driving Data Classification Results

**Figure 3:**
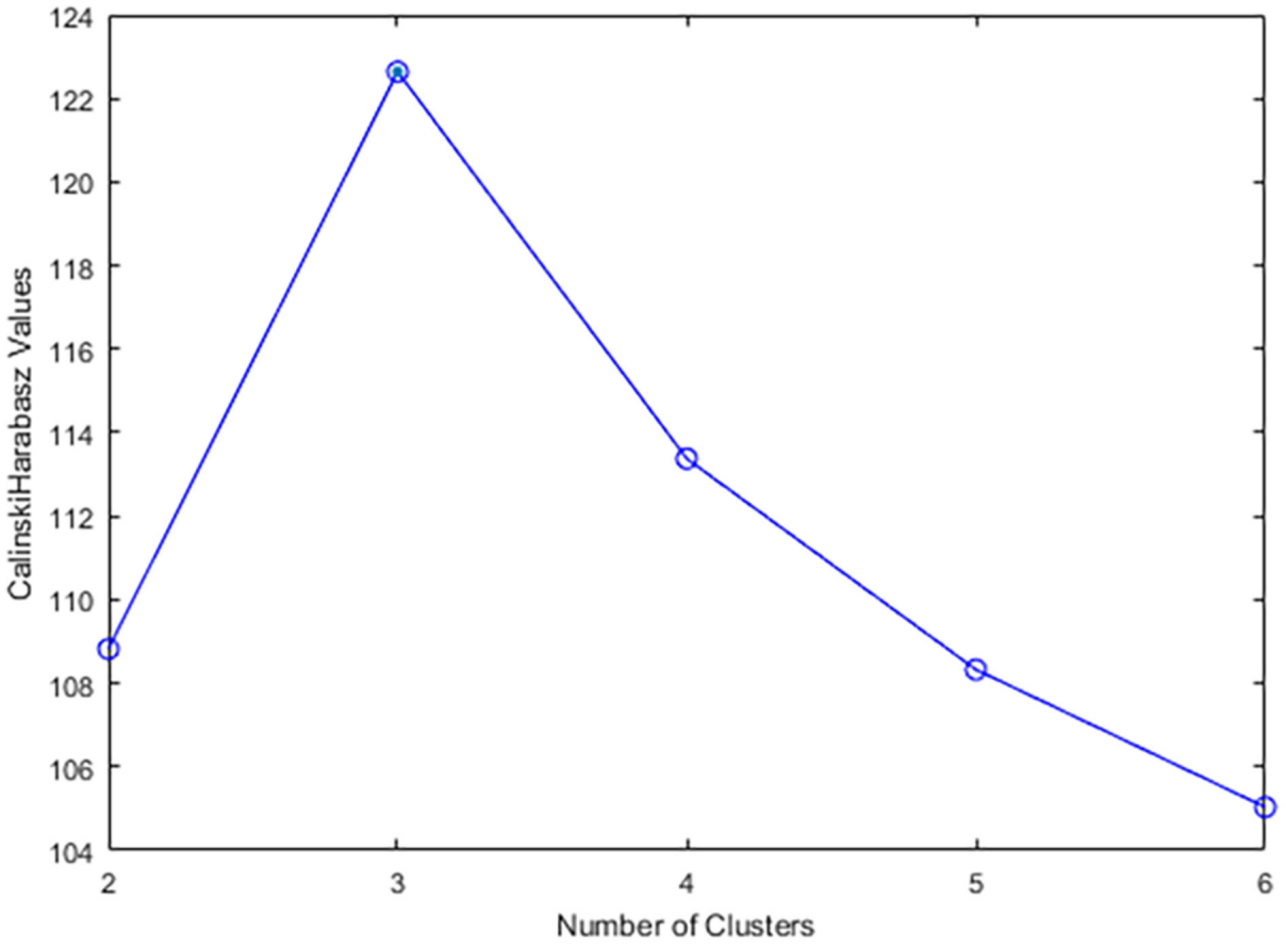
Calinski-Harabasz score corresponding to different number of clusters.

**Figure 4:**
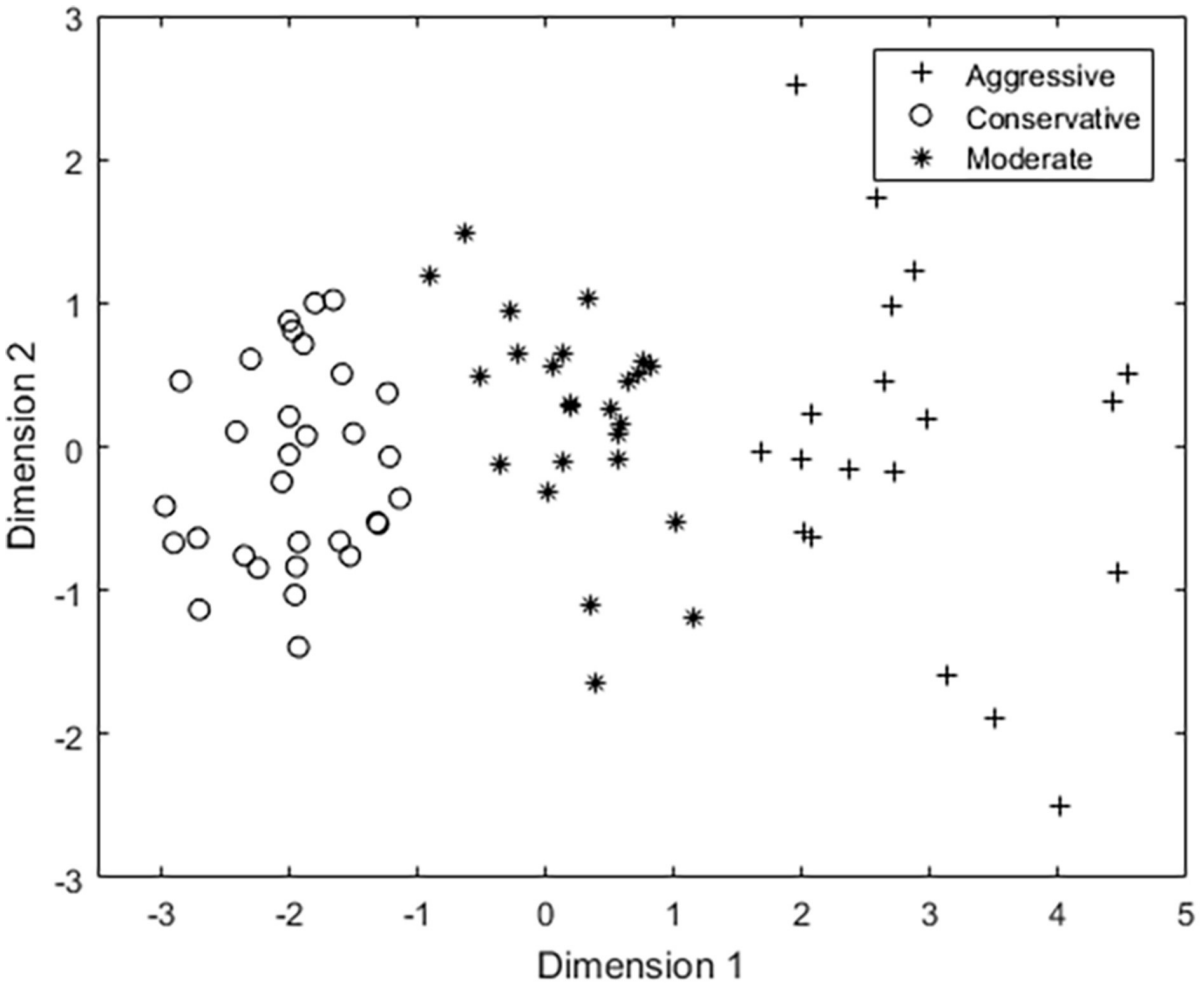
Results of K-means based on the driving data.

No instances of simulator sickness were observed in our experiments. The 23 participants completed 75 driving tasks and hence 75 samples of driving data and EEG data were acquired. The 7-dimension feature vectors of the driving data, i.e., steering wheel rotation angle, angular velocity, angular acceleration, total driving time, vehicle velocity, the number of collisions and the number of lane excursions, were calculated and processed by PCA and reduced to 2-dimensions. The Calinski-Harabasz score was utilized to determine the optimal number of clusters, which was 3 for our dataset (Figure 3). In addition, previous studies have suggested that driving style can be classified into Aggressive type, Moderate type, and Conservative type (Chu et al., 2017; Deng et al., 2017; Li et al., 2017; Palat et al., 2019), accordingly in this paper ***K*** is 3. Three random samples were selected as the initial clustering centroids and the samples were clustered into three driving style groups via the K-means algorithm (Figure 4).

The mean values and standard deviations of the driving data for each group were calculated (Table 1) and the three groups were referred to as the Aggressive group, Moderate group and Conservative group. The analysis of variance (ANOVA) indicated that there was significant difference of the driving data among three groups of different driving styles (Table 1, all *P* < 0.01). The pairwise differences were all significant. The Aggressive group had the most accidents and aberrations including lane excursion and collision, and the largest rotation angle, angular velocity, angular acceleration of the steering wheel and faster vehicle velocity. The Conservative group had the least number of accidents and aberrations, and the smallest rotation angle, angular velocity, angular acceleration, and the vehicle velocity. The Moderate group had mid-level parameters between the Aggressive and Conservative groups.

**Table 1:** Driving variables of the three groups.

**Driving variable**	**Aggressive group (*n* = 19)**	**Moderate group (*n* = 25)**	**Conservative group (*n* = 31)**	**F/P/ η^2^**	**Aggressive vs. moderate**	**Aggressive vs. conservative**	**Moderate vs. conservative**
Velocity(Km/h)^**^	68.2 ± 4.1	62.2 ± 3.3	51.4 ± 2.1	118.2/0.000/0.77	0.000	0.000	0.000
Total time of driving(s)^*^	430.3 ± 18.6	428.3 ± 15.2	499.5 ± 9.6	7.0/0.002/0.15	0.598	0.001	0.000
The number of lane excursions^**^	10.0 ± 4.4	4.8 ± 3.0	1.8 ± 2.1	40.9/0.000/0.53	0.000	0.000	0.000
The number of collisions^**^	4.6 ± 1.9	2.8 ± 1.5	0.7 ± 0.9	47.6/0.000/0.57	0.001	0.000	0.000
Angular velocity of steering wheel(rad/s)^**^	1.96 ± 0.38	1.49 ± 0.18	1.15 ± 0.19	61.8/0.000/0.63	0.000	0.000	0.000
Angular acceleration(rad/s^2^)^**^	1001.1 ± 167.5	697.6 ± 148.7	413.5 ± 118.8	101.8/0.000/0.74	0.000	0.000	0.000
Rotation angle of the steering wheel(°)^**^	48.8 ± 12.6	32.2 ± 5.7	26.2 ± 2.5	57.6/0.000/0.62	0.000	0.000	0.000

* P < 0.01

** P < 0.001

We compared the clustering of driving behavior results with the questionnaire results and the driving style labels of 49 samples were consistent with the participants' self-reports, including 11 in the Aggressive group, 18 in the Conservative group and 20 in the Moderate group.

### EEG Characteristics of Three Groups With Different Driving Styles

**Figure 5:**
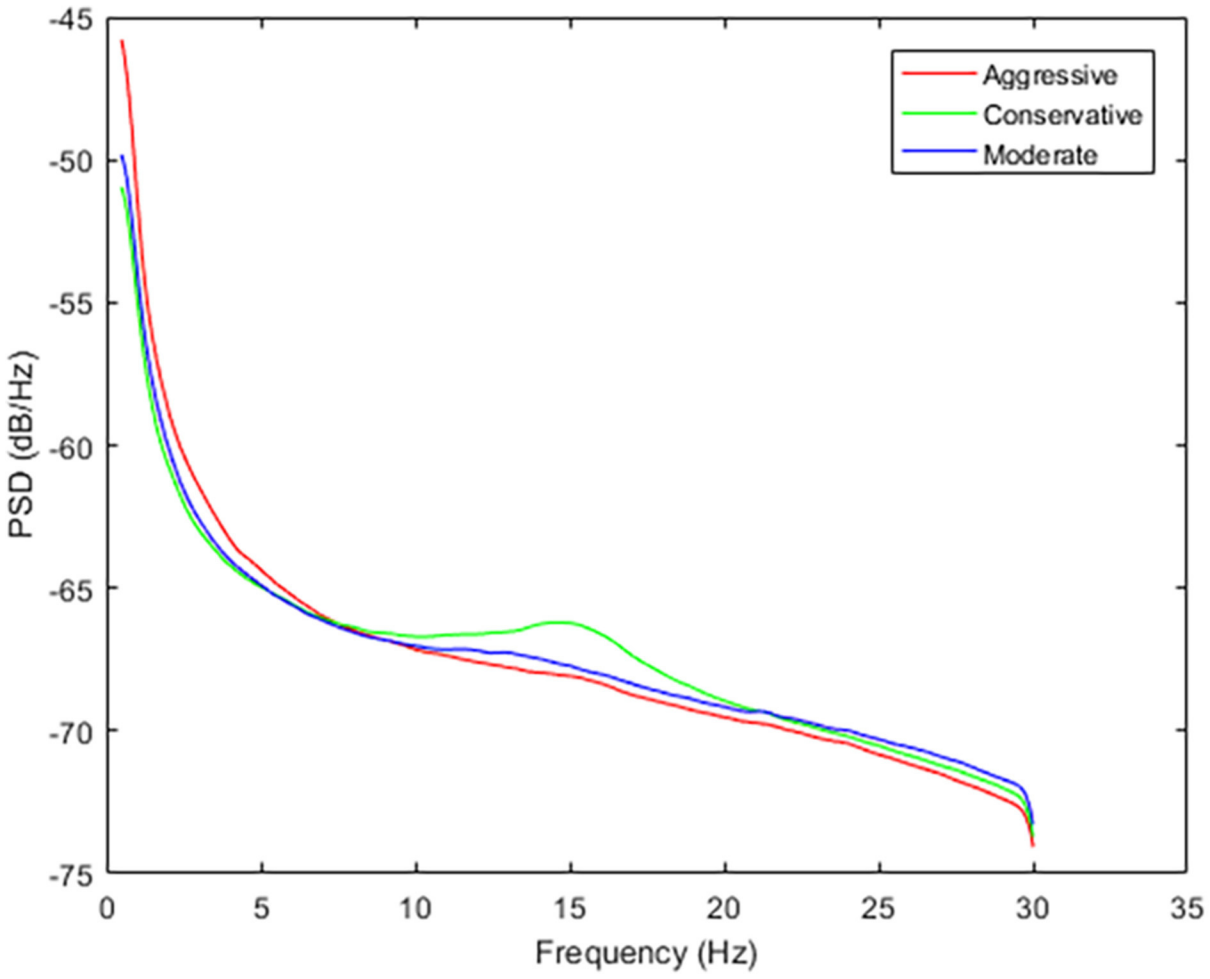
Power spectrum of different driving styles. An obvious bump can be observed in the Conservative group around 15Hz.

The averaged PSDs of EEG data in each group are shown in Figure 5. Generally, the PSDs in all groups decreased with an increase of the frequency, except for the Conservative group, where there was an obvious bump around 15 Hz. The PSD was the highest in the Aggressive group and lowest in the Conservative group, i.e., Aggressive group > Moderate group > Conservative group between 0.5–7 Hz (Band 1), and Conservative group > Moderate group > Aggressive group between 7–21 Hz (Band 2), and Moderate group > Conservative group >Aggressive group between 21–30 Hz (Band 3).

**Figure 6:**
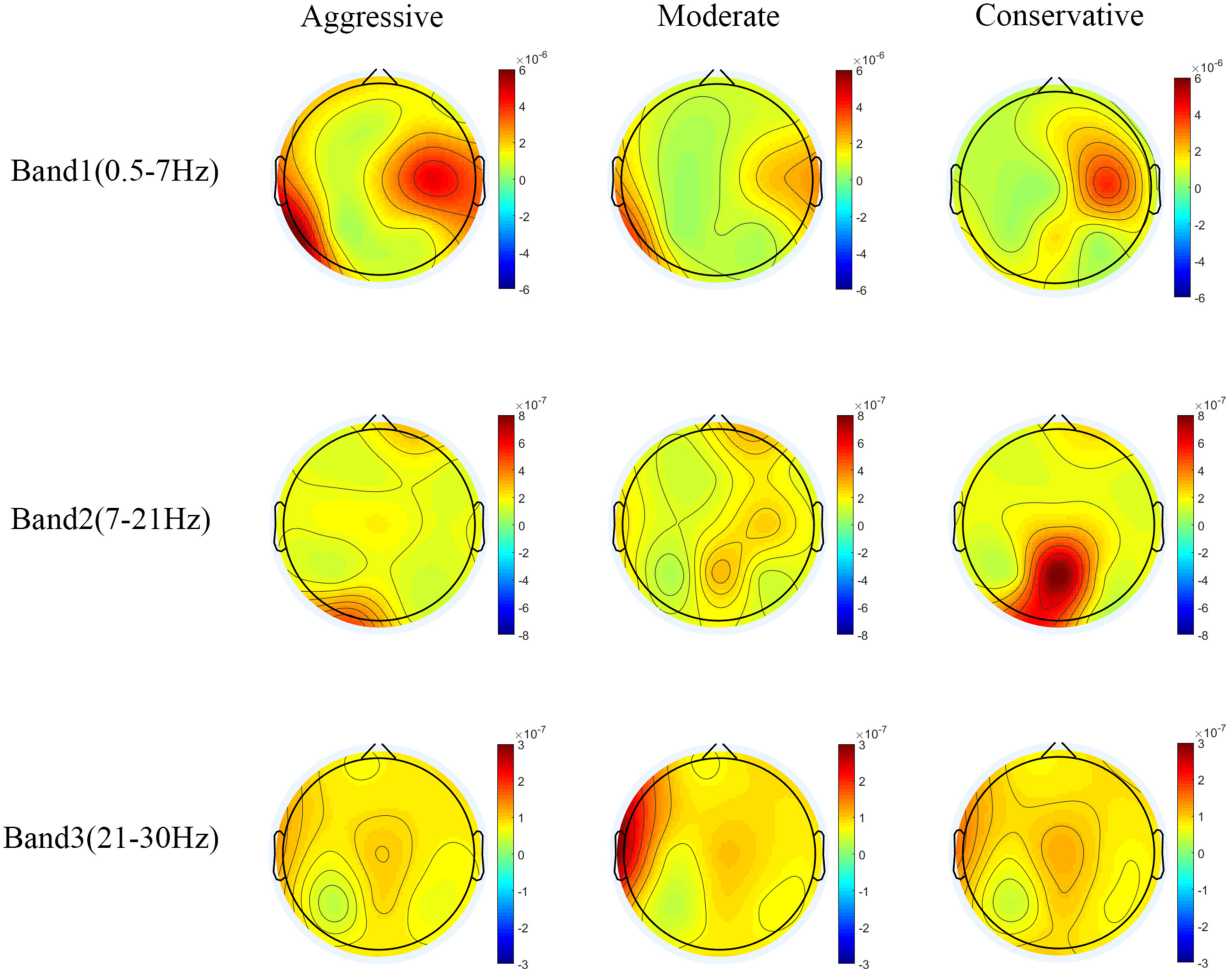
Power topographic maps for the three driving styles in four bands.

The detailed PSD information of all the electrodes in these bands in the three groups are listed in Table 2 and the scalp topography is shown in Figure 6. In Band 1, PSDs were significantly different among the three groups in the parietal (all *P* < *0.05*, η^2^ > 0.11), temporal (all *P* < *0.05*, η^2^> 0.10) and left frontal areas (*P* < 0.01, η^2^ > 0.37). The Aggressive group had higher Band 1 power density in the parietal (except C4, all *P* < *0.05*), temporal (all *P* < *0.05*) and the left frontal areas (P < 0.01) compared with Conservative group. There existed PSD difference in the parietal (all *P* < *0.05*) and left frontal areas (*P* < 0.01) between the Aggressive and Moderate groups. In Band 2, PSDs were significantly different among the three groups in the parietal (all *P* < *0.05*, η^2^ > 0.11) and occipital areas (*P* < *0.05*, η^2^ > 0.11). The Conservative group had the significantly highest PSD values. In band 3, PSDs were significantly different among the three groups in left temporal area (*P* < 0.01, η^2^ = 0.13), and the Moderate group clearly had the significantly highest PSD values.

**Table 2:** Power spectral densities of three groups in three frequency bands.

**Band**	**Channel**	**Aggressive**	**Moderate**	**Conservative**	**F/P/η^2^**	**Aggressive vs. moderate**	**Aggressive vs. conservative**	**Moderate vs. conservative**
		**(dB)**	**(dB)**	**(dB)**				
		**($\left(\bar{x}\;\pm \;s,n=19\right)$**	**($\left(\bar{x}\;\pm \;s,n=25\right)$**	**($\left(\bar{x}\;\pm \;s,n=31\right)$**				
Band 1	Fz	−60.7 ± 2.1	−62.6 ± 1.4	−58.1 ± 4.1	1.6/0.20/0.04	-	-	-
	F8	−59.4 ± 2.3	−59.1 ± 3.1	−61.6 ± 1.8	0.7/0.51/0.02	-	-	-
	Cz	−56.9 ± 4.0	−61.8 ± 1.5	−62.6 ± 1.3	13.0/0.00/0.26	0.00	0.00	0.15
	Pz	−59.5 ± 2.7	−61.2 ± 2.5	−57.6 ± 4.1	0.5/0.60/0.01	-	-	-
	T6	−56.3 ± 4.1	−58.7 ± 3.1	−59.7 ± 2.8	2.1/0.14/0.05	-	-	-
	T5	−51.9 ± 5.7	−54.4 ± 4.7	−58.3 ± 2.4	3.7/0.03/0.10	0.27	0.02	0.01
	C4	−53.3 ± 4.8	−56.8 ± 3.9	−54.0 ± 5.1	4.6/0.01/0.11	0.00	0.75	0.31
	C3	−60.1 ± 2.7	−62.9 ± 1.5	−63.2 ± 1.1	9.5/0.00/0.21	0.01	0.00	0.40
	T4	−54.9 ± 3.9	−55.6 ± 4.2	−61.4 ± 1.7	4.3/0.02/0.11	0.84	0.00	0.27
	T3	−54.4 ± 4.7	−55.9 ± 3.3	−60.7 ± 2.3	5.9/0.00/0.14	0.34	0.00	0.01
	O2	−59.8 ± 2.3	−61.9 ± 1.6	−61.9 ± 1.7	1.3/0.28/0.03	-	-	-
	O1	−57.8 ± 2.7	−59.2 ± 1.8	−58.5 ± 3.0	0.6/0.55/0.02	-	-	-
	P4	−59.0 ± 3.3	−62.4 ± 1.9	−63.2 ± 1.4	13.2/0.00/0.27	0.04	0.00	0.19
	P3	−61.4 ± 2.9	−63.9 ± 1.8	−63.7 ± 2.3	4.7/0.01/0.12	0.01	0.02	0.79
	Fp2	−58.0 ± 2.6	−60.0 ± 2.6	−60.3 ± 2.2	1.0/0.38/0.03	-	-	-
	Fp1	−57.0 ± 2.5	−60.5 ± 2.1	−61.4 ± 1.2	21.1/0.00/0.37	0.00	0.00	0.19
Band 2	Fz	−68.5 ± 0.5	−68.6 ± 0.2	−68.2 ± 0.6	1.1/0.34/0.03	-	-	-
	F8	−68.4 ± 0.5	−68.3 ± 0.7	−68.3 ± 0.5	0.2/0.79/0.008	-	-	-
	Cz	−67.0 ± 1.1	−67.4 ± 0.2	−65.8 ± 1.8	5.5/0.01/0.13	0.04	0.11	0.00
	Pz	−67.9 ± 1.3	−65.3 ± 3.0	−60.8 ± 3.5	4.5/0.01/0.11	0.47	0.00	0.00
	T6	−68.7 ± 1.0	−68.6 ± 0.2	−68.3 ± 0.6	2.1/0.13/0.06	-	-	-
	T5	−67.5 ± 1.0	−67.6 ± 0.6	−67.7 ± 0.8	0.8/0.46/0.02	-	-	-
	C4	−68.2 ± 0.9	−65.9 ± 3.1	−67.4 ± 1.9	1.6/0.22/0.04	-	-	-
	C3	−68.2 ± 2.0	−68.6 ± 0.3	−67.5 ± 1.7	1.1/0.35/0.03	-	-	-
	T4	−67.8 ± 1.3	−68.0 ± 1.1	−68.2 ± 0.5	0.8/0.46/0.02	-	-	-
	T3	−67.8 ± 0.6	−66.2 ± 2.0	−67.7 ± 1.4	0.9/0.42/0.02	-	-	-
	O2	−68.1 ± 0.5	−68.1 ± 0.2	−68.0 ± 0.4	0.4/0.64/0.01	-	-	-
	O1	−63.5 ± 3.7	−68.2 ± 0.3	−62.4 ± 3.3	5.2/0.01/0.13	0.26	0.00	0.00
	P4	−69.3 ± 0.5	−69.0 ± 1.2	−66.8 ± 2.7	2.1/0.13/0.06	-	-	-
	P3	−69.2 ± 2.9	−71.9 ± 0.4	−68.1 ± 3.4	1.2/0.29/0.03	-	-	-
	Fp2	−65.4 ± 3.0	−65.2 ± 3.2	−66.2 ± 2.6	0.2/0.84/0.005	-	-	-
	Fp1	−68.3 ± 0.4	−68.4 ± 0.2	−68.2 ± 0.5	1.2/0.32/0.03	-	-	-
Band 3	Fz	−70.8 ± 0.1	−70.7 ± 0.2	−70.5 ± 0.5	5.0/0.01/0.12	0.02	0.01	0.07
	F8	−70.7 ± 0.1	−70.6 ± 0.2	−70.6 ± 0.4	1.2/0.30/0.03	-	-	-
	Cz	−69.9 ± 0.2	−69.8 ± 0.2	−69.3 ± 1.0	7.1/0.00/0.17	0.30	0.01	0.01
	Pz	−70.4 ± 0.7	−70.6 ± 1.2	−70.3 ± 1.5	0.4/0.66/0.01	-	-	-
	T6	−71.7 ± 0.9	−71.4 ± 0.3	−71.3 ± 0.6	2.7/0.08/0.07	-	-	-
	T5	−70.8 ± 0.5	−70.7 ± 0.4	−70.9 ± 0.6	0.5/0.58/0.02	-	-	-
	C4	−71.2 ± 0.5	−70.9 ± 1.1	−71.1 ± 0.8	1.2/0.32/0.03	-	-	-
	C3	−71.5 ± 0.5	−71.6 ± 0.1	−71.3 ± 0.6	2.1/0.13/0.06	-	-	-
	T4	−71.2 ± 0.2	−70.9 ± 0.6	−71.0 ± 0.7	0.5/0.59/0.01	-	-	-
	T3	−69.0 ± 0.4	−66.8 ± 3.4	−68.8 ± 1.9	5.0/0.01/0.13	0.02	0.47	0.03
	O2	−71.1 ± 0.1	−71.0 ± 0.2	−70.9 ± 0.5	1.0/0.37/0.03	-	-	-
	O1	−71.2 ± 0.8	−71.2 ± 0.3	−70.6 ± 1.3	4.7/0.01/0.11	0.76	0.06	0.01
	P4	−72.0 ± 0.1	−71.7 ± 1.0	−71.5 ± 1.0	1.4/0.25/0.04	-	-	-
	P3	−74.9 ± 0.6	−74.9 ± 0.4	−74.4 ± 1.3	3.1/0.04/0.08	0.90	0.10	0.04
	Fp2	−71.0 ± 0.7	−70.7 ± 0.7	−70.8 ± 0.8	0.8/0.45/0.02	-	-	-
	Fp1	−71.4 ± 0.1	−71.4 ± 0.1	−71.3 ± 0.4	0.2/0.81/0.006	-	-	-

*ANOVA among the three groups was performed. If P < = 0.05, then the pairwise comparison (LSD) among three groups was performed*.

### EEG Data Classification Results

The original 8-dimension EEG feature vectors were reduced to 2-dimensions by using the LDA method, and then used as the input data to train the SVM model. The classification performance evaluated by the leave-one-subject-out cross validation approach is listed in Table 3. The overall accuracy was 80.0%, the precision and recall for the Aggressive group were 83.3 and 78.9% respectively, for the Moderate group 70.0 and 84.0%, respectively, and for the Conservative group 88.9 and 77.4%, respectively. The F-measures of the Aggressive group, the Moderate group and the Conservative group were 81.0, 76.4, and 82.8% respectively.

**Table 3:** Confusion matrix of the SVM model.

**Accuracy**	**80.0%**	**True label**			**Precision**
		**Aggressive driving style**	**Moderate driving style**	**Conservative driving style**	
Predicted label	Aggressive driving style	**15**	2	1	83.3%
	Moderate driving style	3	**21**	6	70.0%
	Conservative driving style	1	2	**24**	88.9%
Recall		78.9%	84.0%	77.4%	

*Bold values indicate that the true label is consistent with the predicted label*.

We compared the SVM classification results with the questionnaire results. The driving style labels based on SVM of 47 samples were consistent with the participants' self-reports, including 9 in the Aggressive group, 16 in the Conservative group and 22 in the Moderate group.

## Discussion

In this study we presented a driving style recognition schema based on a combination of EEG and driving behavioral data. The driving data included the velocity, the total driving time, the number of lane excursion, the number of collision, the rotation angle, the angular velocity and the angular acceleration of the steering wheel, which mainly reflected the driving behavior of the participants. EEG data mainly reflected the cognitive status of the participants during driving. The driving data was clustered into three clusters using the *K*-means algorithm, which corresponded to three driving styles, i.e., Aggressive, Moderate and Conservative. The EEG features in the frequency domain, including the amplitude and the PSDs of the EEG signal in δ, θ, α, and β bands, along with the cluster results of the driving data, were utilized to train the SVM classification model. The leave-one-subject-out cross validation approach showed considerable classification performance of the schema with the total accuracy of 80.0%, the highest precision 88.9% and the highest recall 84.0%. The *F*-measures showed that this classifier was approximately equally sensitive to the three driving styles and the classification performance was balanced. These results suggested a close relationship between EEG and driving style and demonstrated the feasibility of driving style recognition and prediction using EEG data.

### Relationship of Driving Behavior and Driving Style

Large mean values of the driving behaviors indicated the driver's preference for speeding and turning sharply and quickly, which meant the driver was inclined to an “Aggressive driving style,” whereas small mean values indicated the driver's preference for keeping a low speed and turning the steering wheel conservatively, which meant the driver was inclined to a “Conservative driving style.” As shown in Table 1, steering wheel rotation angle, angular velocity, angular acceleration, total driving time, vehicle velocity, and number of collisions and number of lane excursions were all the highest in the Aggressive group and lowest in the Conservative group. Moreover, the number of accidents and aberrations increased with the agressiveness of the driving style. Consistent with previous studies (Reason et al., 1990; Martinussen et al., 2014; Lee and Jang, 2017), these results demonstrate the close relationship between driving behavior characteristics and driving styles.

Previous studies have regarded a driver's driving style as fixed and difficult to change (Chen et al., 2013; Shi et al., 2015). However, in this paper, we found that 13 participants maintained the driving style during the whole experiment, 4 participants' driving styles varied between conservative and moderate, 4 participants' driving styles varied between aggressive and moderate, and 2 participants' driving styles varied between aggressive and conservative. These results indicate that a driver's driving style may fluctuate to some extent.

Driving skill refers to how good a person is at handling a car, and it is typically measured by the standard deviation of the driving data, which is negatively correlated with the stability of the driving skill (Lu, 2011; Martinussen et al., 2014). As shown in Table 1, the standard deviations for almost all driving variables were Aggressive group > Moderate group > Conservative group, which indicated that driving skill may have a potential relationship with the driving style. The more aggressive the driving style, the more variable the driving skills.

### Relationship of EEG Characteristics and Driving Style

In Band 1 (0.5–7 Hz), the Aggressive group had significantly higher PSD values in the left temporal area than the Conservative group (Figure 6), which meant more delta and theta power in the temporal gyrus of aggressive drivers, which was related with more emotion fluctuations when driving (Knyazev et al., 2009). As shown in Figure 5, in the theta band (4–7 Hz), the Aggressive group had the highest PSD among the three groups. While in the beta band (13–30 Hz), the Aggressive group had the lowest PSD. Moreover, the Aggressive group had the highest PSD in the frontal area in Band 1 (Figure 6, Table 2). These results indicate that the Aggressive group had highest theta/beta ratio in the frontal area compared with the other two groups, which implies that aggressive drivers had poorer executive cognitive control and attentional control (Angelidis et al., 2016, 2018), and might have greater mental workload (Matthews et al., 2017; Karthaus et al., 2018; Puma et al., 2018). In Figure 5, it can be seen that the Conservative group's PSD increased along with an increase of in frequency and was the highest in the alpha band (7–13 Hz), which possibly implies that conservative drivers had a more relaxed mental state (Karthaus et al., 2018). In beta 1 (13–18Hz) and beta 2 (18–21Hz), the Conservative group's PSD was the highest (Figure 5) and concentrated over the parietal area which was related with associate sensory function(Tao et al., 2010) (Figure 6). It seems that conservative drivers were more inclined to the pro-active driving state (Garcia et al., 2017), which was associated with a better anticipation and active use of ongoing information, and a more proactive planning of future responses (Getzmann et al., 2018). According to the above analysis based on EEG signals, conservative drivers were less likely to have aberrational driving behaviors like “violations” and “errors” (Reason et al., 1990).

### Novelty and Limitations of This Study

Prior studies utilized questionnaires and/or objective driving behavior data to recognize driving styles (Ly et al., 2013; Martinussen et al., 2014; Hooft van Huysduynen et al., 2018). Different from these studies, we developed a driving style recognition schema based on the combination of objective driving data and a psychophysiological signal—EEG data. The objective behavior driving data is a direct reflection of the driving behavior, which is associated with the driver's brain activity and cognitive state. The traditional questionnaire is a subjective and indirect reflection of the human cognitive trait. Furthermore, because its measurement would occupy the full attention or interrupt the normal activity of the driver, it can't be applied to evaluate the driver's driving style in real time without interference. In contrast, EEG data is a direct reflection of the underlying cognitive state. Except for the requirement of wearing an electrode cap, there is not much interference with the behavior of the participants. Besides, EEG is the objective evaluation, which is less likely to be affected by the subjective factors of the experimenters and the participants (Taubman-Ben-Ari et al., 2004; Martinussen et al., 2014). Accordingly, the results could be more reliable and comparisons among different studies would be more feasible. EEG has high temporal resolution, which is at the same time scale as the underlying mental activity, so it can be applied on the real-time online occasions in the future. Considering that driving is a time-varying behavior, prediction and intervention of dangerous behaviors requires the system to have high temporal performance. Thus, it is of great practical significance to use EEG to identify the driving style and to warn the drivers of dangerous behaviors. Generally, our schema of simultaneous collecting and unified analysis of the driving and the EEG data from a simulated driving system provided a new method for driving style recognition.

Driving style recognition plays a significant role in the ADAS, which could help to identify the current status of the driver and adjust the vehicle parameters accordingly to ensure safe driving. As shown in Table 1 and demonstrated by previous studies, drivers with an Aggressive style tend to operate the vehicle intensively and cause more accidents (Yang et al., 2018), so this driving style is regarded as unsafe and should to be avoided. But it is noted that an Aggressive driving style does not inevitably result in dangerous behaviors (Taubman-Ben-Ari et al., 2004; Yang et al., 2018). What is more important, brain activities are the preconditions of the behaviors, and usually precede the actual behaviors. By using our schema, the dangerous driving style related real-time EEG features could be monitored and detected. And then the driving assistance system can initiate a warning procedure immediately by reminding the driver to adjust his/her behavior, or even take over the vehicle by adjusting the controlling parameters of the steering wheel and the accelerator pedal. These actions could avoid the occurrence and diminish the adverse consequences of dangerous driving behaviors.

Driving style recognition methods can also be utilized to improve driving experience and comfort. Previous research suggested that a driver may exhibit different driving styles in different traffic conditions (Yang et al., 2018). This variability was also observed in our results. By integrating our schema with the driving assistance system, multiple sets of driving parameters can be set for different driving styles and individualized for different drivers according to their daily driving behaviors and his/her own preferences. What's more, EEG data can reflect the driver's physiological state, such as fatigue and distraction (Wang et al., 2015; Hajinoroozi et al., 2016; Guo et al., 2018; Ma et al., 2018).

There are some limitations of this study. The traffic scenario was relatively simple without considering multiple driving scenarios, such as traffic jams. The changes of driving style under different driving scenarios should be analyzed in future. The complexity of the scenario would affect the degree of driving difficulty. Specifically the performance of turning was related to different driving styles (Ly et al., 2013; Choi et al., 2017; Deng et al., 2018) and brain dynamics (Garcia et al., 2017). Hence in this study, a curved mountainous road was chosen as the scenario. The participants reported difficulty in driving in this scenario and their performance differed among groups with different driving styles. The cognitive load and simulator related side effects were not considered and this is a limitation of our study. The relationship and differentiation between driving style and driving ability, and the manifestation in EEG signals are worthy of further analysis. Other kinds of scenarios, or the available control scenarios should be studied further. The participants may have been biased because of their young ages and short driving years, and the unbalanced male and female ratio. Because the driving styles were divided based on the task-specific data instead of the subject-specific data, the impact of the demographic characteristics of the participants on the driving styles could not be analyzed by using the current schema, which is worthy of further analysis. During the experiment some participants reported fatigue and expressed their will to terminate the driving tasks. Hence, the number of the tasks performed by each participant varied between two to four. How to improve the experiment and how to diminish the impact of task number inconsistencies among different participants warrant further research. The presented schema was offline, which needs to be improved to fulfill the requirement of online analysis. Its performance under a realtime condition warrants further research. Finally, because of the limitation of the simulated driving experiments, the driver's perception of the surroundings, the vehicles and the roads may be biased, so actual driving experiments need to be conducted in the future studies.

## Ethics Statement

This study was carried out in accordance with the recommendations of the ethical review committee of Wuhan University of Technology with written informed consent in accordance with the Declaration of Helsinki from all participants. The protocol was approved by the ethical review committee of Wuhan University of Technology.

## Author Contributions

FY and ML designed the data processing schema and wrote the manuscript. ML, CD, and YW designed the experiment and were involved in the data collection. LY conceived the basic frame of this study and revised the manuscript.

### Conflict of Interest Statement

The authors declare that the research was conducted in the absence of any commercial or financial relationships that could be construed as a potential conflict of interest.
